# Prefrontal Oxygenation During Exercise and Inhibitory Control After Aerobic and Game-Based Exercise in Young Adults

**DOI:** 10.3390/brainsci16060558

**Published:** 2026-05-23

**Authors:** Youmin Son, Yeonhak Jung

**Affiliations:** 1 Department of Physical Education, Korea University, Seoul 02841, Republic of Korea; domkoi85@gmail.com; 2 Department of Kinesiology, California State University, Northridge, CA 91330, USA

**Keywords:** acute exercise, inhibitory control, fNIRS, prefrontal cortex, game-based exercise, cognitively engaging exercise, executive function, young adults

## Abstract

**Highlights:**

**What are the main findings?**

- Greater global prefrontal oxy-Hb during exercise was associated with greater subsequent improvement in inhibitory control, indexed by reduced Flanker cost.
- Game-based exercise elicited higher prefrontal oxy-Hb during exercise than aerobic exercise, but Flanker cost improvement did not differ significantly between conditions.

**What are the implications of the main findings?**

- Prefrontal oxygenation during exercise may help explain individual differences in post-exercise cognitive benefits.
- Game-based exercise may provide a useful model for studying how cognitively engaging movement influences brain oxygenation and executive function.

**Abstract:**

**Background/Objectives**: Acute exercise can influence executive function, but the neurophysiological responses linking exercise to cognitive change remain unclear. Functional near-infrared spectroscopy (fNIRS) provides a feasible method for assessing prefrontal oxygenation during movement-based exercise. This study examined whether prefrontal oxygenated hemoglobin (oxy-Hb) during exercise was associated with subsequent changes in inhibitory control after aerobic and game-based exercise in young adults. **Methods**: Twenty-four healthy young adults completed aerobic and game-based exercise conditions in a randomized, counterbalanced, within-subject design. The aerobic condition consisted of jogging, whereas the game-based condition consisted of a pickleball-based activity. Exercise intensity was monitored during both conditions. Prefrontal oxy-Hb was recorded during exercise using fNIRS, and inhibitory control was assessed before and after each condition using an Eriksen Flanker task. The primary behavioral outcome was Flanker cost improvement, and the primary fNIRS outcome was mean baseline-corrected prefrontal oxy-Hb during exercise. **Results**: Exercise intensity was comparable between conditions. Greater mean prefrontal oxy-Hb during exercise was significantly associated with greater improvement in Flanker cost (β = 3.71 ms per 0.01 μM, 95% CI [2.13, 5.30], *p* < 0.001). Game-based exercise elicited a higher mean prefrontal oxy-Hb during exercise than aerobic exercise. No significant condition difference was observed for Flanker cost improvement. **Conclusions**: Prefrontal oxygenation during exercise was associated with subsequent improvement in inhibitory control. These findings suggest that neurophysiological responses during exercise may account for some between-person variability in acute exercise-related cognitive benefits.

## 1. Introduction

Acute exercise can transiently influence executive function. However, the magnitude and direction of this effect are not uniform and may depend on exercise intensity, exercise modality, participant characteristics, cognitive domain, and the timing of post-exercise assessment [1,2,3]. Among executive function domains, inhibitory control is particularly relevant because it reflects the ability to suppress irrelevant or inappropriate responses and to resolve response conflict. The Eriksen Flanker task is widely used to assess inhibitory control by comparing performance on congruent and incongruent trials [4,5], with the Flanker cost reflecting the behavioral interference produced by distracting flankers. Previous studies have shown that a single bout of moderate-to-vigorous aerobic exercise may improve inhibitory control in young adults; however, these effects are not consistently observed and appear to vary depending on the exercise protocol, timing of assessment, and outcome measure [1,2,3]. These mixed findings suggest that acute exercise effects on inhibitory control are likely context-dependent and may require simultaneous consideration of behavioral and neurophysiological responses.

Several mechanisms have been proposed to explain the acute exercise–cognition relationship. These include changes in arousal, exercise-related neurochemical activity, cerebral blood flow and oxygenation, lactate-related metabolic signaling, and neurotrophic factors such as brain-derived neurotrophic factor (BDNF) [6,7]. Although these mechanisms are not mutually exclusive, it remains unclear which physiological responses are most closely linked to short-term changes in executive function. In this context, cerebral oxygenation has received increasing attention because it provides a plausible bridge between exercise-induced physiological activation and cognitive performance. Functional near-infrared spectroscopy (fNIRS) offers a non-invasive and relatively ecologically valid method for monitoring prefrontal hemodynamic responses during both physical activity and cognitive task performance [8].

The prefrontal cortex (PFC) plays an important role in inhibitory control, conflict monitoring, attentional regulation, and flexible allocation of cognitive resources [9]. fNIRS is particularly useful in exercise–cognition research because it allows cortical oxygenation to be assessed during movement-based protocols that are difficult to examine using more motion-sensitive neuroimaging techniques [8]. Oxygenated hemoglobin (oxy-Hb) is commonly used as an index of cortical oxygenation and task-related hemodynamic response. However, oxy-Hb during exercise must be interpreted cautiously because it may be influenced not only by neural activity but also by systemic physiological changes, including heart rate, respiration, blood pressure, scalp blood flow, and motion-related artifacts [8,10,11]. Therefore, higher oxy-Hb during exercise should not be interpreted automatically as greater or more beneficial neural activation.

In addition to physiological intensity, the cognitive demands embedded within exercise may be important for understanding exercise-related cognitive outcomes. Traditional aerobic exercise, such as jogging, primarily emphasizes metabolic and cardiovascular load and generally involves relatively predictable, repetitive movement patterns characteristic of closed-skill exercise [12,13,14,15]. In contrast, game-based exercise can be conceptualized as a cognitively engaging exercise modality because it integrates physical exertion with continuous perceptual, decision-making, and motor-coordination demands [12,13,14,15]. This perspective is consistent with the argument that exercise–cognition research should move beyond quantitative characteristics such as intensity and duration alone and consider qualitative characteristics of movement, including cognitive challenge, task complexity, and cognitive–motor integration [13,14,15].

Within this framework, game-based exercise can be characterized as a cognitively engaging modality with greater cognitive–motor demands than relatively repetitive aerobic exercise. This characterization reflects the broader idea that movement requiring decision-making, visuomotor coordination, and adaptation to changing environmental demands may recruit executive control processes during exercise [12,13,14,15]. Importantly, this distinction does not imply that aerobic exercise is cognitively passive; rather, it differentiates exercise modalities according to the degree of embedded cognitive engagement and environmental complexity.

Emerging evidence suggests that open-skill or cognitively engaging exercise may have distinct effects on executive function compared with closed-skill aerobic exercise. For example, an acute fNIRS study reported that open-skill exercise improved inhibitory control without a corresponding increase in task-related prefrontal activation during subsequent cognitive testing, suggesting that behavioral improvement may sometimes reflect more efficient neural processing rather than greater cortical recruitment [16]. This finding is important because it cautions against interpreting larger oxy-Hb responses as inherently more beneficial. Accordingly, the mechanistic question in the present study is not simply whether game-based exercise elicits greater prefrontal oxygenation than aerobic exercise, but whether individual differences in prefrontal oxy-Hb during exercise are associated with subsequent changes in inhibitory control.

Despite growing interest in cognitively engaging and open-skill exercise as modalities that may differentially influence executive function [12,13,14,15], the neurophysiological basis of these effects remains insufficiently characterized. Although fNIRS has been increasingly used to examine prefrontal hemodynamic responses in exercise–cognition research [8], few acute studies have directly compared aerobic or closed-skill exercise with cognitively engaging or game-based exercise while simultaneously assessing behavioral performance, prefrontal oxygenation, and exercise intensity within the same experimental framework [16].

This gap is important because modality-related differences in cognitive performance or prefrontal oxy-Hb may be misinterpreted if physiological intensity is not adequately monitored. For example, a game-based activity may appear to elicit greater prefrontal oxy-Hb because of greater cardiovascular or movement-related demands rather than cognitive engagement itself. To address this issue, the present study monitored heart rate, accelerometry, and ratings of perceived exertion to determine whether aerobic and game-based conditions were performed at comparable intensity.

The present study examined whether prefrontal oxy-Hb during exercise is associated with subsequent changes in inhibitory control following intensity-monitored aerobic and game-based exercise in young adults. Aerobic exercise was represented by jogging, whereas game-based exercise was represented by a pickleball-based activity. Participants completed both conditions in a randomized, counterbalanced, within-subject design. Exercise intensity was monitored using heart rate, accelerometry, and ratings of perceived exertion (RPE). Prefrontal oxy-Hb was recorded during the exercise, and Flanker task performance was assessed before and after each condition.

The primary aim was to determine whether global prefrontal oxy-Hb during exercise was associated with pre-to-post improvement in Flanker cost. Secondary aims were to compare aerobic and game-based exercise on global prefrontal oxy-Hb during exercise and Flanker task outcomes, with exploratory analyses examining regional prefrontal oxy-Hb responses. It was hypothesized that greater global prefrontal oxy-Hb during exercise would be associated with greater subsequent improvement in inhibitory control, reflected by a reduction in Flanker cost. Given its greater cognitive–motor demands, game-based exercise was expected to elicit greater prefrontal oxy-Hb than aerobic; differences in inhibitory control improvement were examined as a secondary aim.

## 2. Materials and Methods

### 2.1. Participants

Twenty-seven healthy young adults aged 18–30 years were enrolled from the university and the surrounding community. Participants were eligible if they were free from known neurological, psychiatric, cardiovascular, or musculoskeletal conditions that could affect exercise participation, cognitive testing, or functional near-infrared spectroscopy (fNIRS) signal quality. Participants were also required to have normal or corrected-to-normal vision and no current medication use known to influence cognitive performance, cerebral hemodynamics, or exercise tolerance.

Physical activity readiness was screened using the Physical Activity Readiness Questionnaire (PAR-Q) [17], and habitual physical activity was assessed using the International Physical Activity Questionnaire (IPAQ) [18]. Participants reporting a potential contraindication to physical activity were excluded or required to provide medical clearance before participation. Participants were instructed to avoid vigorous exercise, alcohol, and caffeine for 24 h before testing and to maintain their usual sleep routine before the laboratory visit. Participants were excluded from the analytic sample if fewer than 70% of fNIRS channels met acceptable signal-quality criteria for condition-level analysis. All procedures were approved by the Institutional Review Board (IRB-FY22-295), and all participants provided written informed consent before participation.

### 2.2. Study Design and Procedure

**Figure 1:**
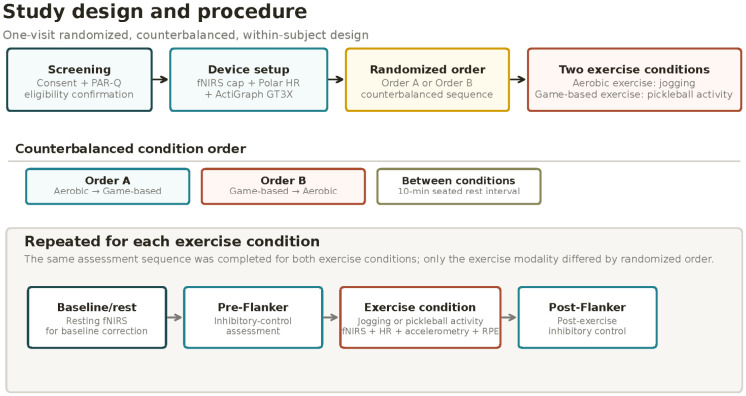
Study design and experimental procedure. Participants completed a one-visit randomized, counterbalanced, within-subject protocol involving aerobic exercise and game-based exercise. After screening, informed consent, PAR-Q eligibility confirmation, and device setup, participants completed both exercise conditions in randomized order. The same assessment sequence was repeated for each condition: baseline/rest fNIRS recording, pre-exercise Flanker task, exercise condition with fNIRS and intensity monitoring, and post-exercise Flanker task. The aerobic condition consisted of jogging, whereas the game-based condition consisted of pickleball activity. Conditions were separated by a 10 min seated rest interval. fNIRS = functional near-infrared spectroscopy; HR = heart rate; RPE = rating of perceived exertion.

This study used a randomized, counterbalanced, within-subject design. Each participant completed one aerobic exercise condition and one game-based exercise condition during a single laboratory visit. The aerobic exercise condition consisted of jogging, whereas the game-based exercise condition consisted of a pickleball-based activity. Condition order was randomized and counterbalanced across participants to reduce order effects (Figure 1).

After informed consent and screening, participants were fitted with the fNIRS cap, and signal quality was checked before data collection. For each condition, participants first completed a seated resting baseline, followed by a pre-exercise Flanker assessment. Participants then completed the assigned exercise condition while fNIRS oxy-Hb, heart rate, and accelerometer data were recorded. Ratings of perceived exertion (RPE) were collected immediately after exercise, and the post-exercise Flanker assessment began within approximately 3–5 min of exercise cessation, with concurrent fNIRS recording. This timing was kept consistent across the aerobic and game-based conditions.

Because both exercise conditions were completed during one visit, a 10 min seated rest interval was included between conditions to reduce immediate carryover effects. This interval length was chosen to balance recovery with the practical constraints of a single-visit protocol and was kept consistent across both exercise conditions. After the rest interval, participants repeated the same sequence for the second condition: resting baseline, pre-exercise Flanker assessment, exercise, RPE assessment, and post-exercise Flanker assessment. A separate resting baseline was recorded before each condition to support condition-specific baseline correction of fNIRS oxy-Hb. The fNIRS cap remained in place throughout the visit, when possible changes in optode placement were minimized.

### 2.3. Exercise Conditions and Intensity Monitoring

The aerobic exercise condition consisted of 10 min of jogging on an indoor gym track at a target light-to-moderate intensity. This condition represented a continuous rhythmic activity with relatively predictable movement demands. The game-based exercise condition consisted of 10 min of pickleball-based activity performed with a research assistant across a net. Participants engaged in repeated ball exchanges, sustained rallies, and brief point-based play, requiring visuomotor tracking, response selection, coordination, and continuous adaptation to a changing task environment. To standardize the pickleball-based condition, all participants played with the same experienced research assistant, who was instructed to maintain continuous rallies rather than competitive match play. The assistant adjusted ball placement and tempo to keep exchanges feasible and engaging, using informal mini-games to provide a consistent task structure while promoting broadly comparable movement and cognitive demands across participants. This approach was intended to provide broadly comparable visuomotor and decision-making demands across participants despite individual differences in skill level.

The two exercise conditions were matched for duration and monitored to promote comparable exercise intensity across conditions. Heart rate was recorded using a Polar H10 heart rate monitor (Polar Electro Oy, Kempele, Finland), and movement intensity was quantified using an ActiGraph GT3X accelerometer (ActiGraph LLC, Pensacola, FL, USA). Mean heart rate, peak heart rate, accelerometer-derived activity counts, and post-exercise RPE on the Borg 6–20 scale were recorded for each condition. These measures were used to evaluate whether the aerobic and game-based conditions were performed at comparable intensity and to inform sensitivity analyses if meaningful intensity differences were observed.

### 2.4. fNIRS Measurement

**Figure 2:**
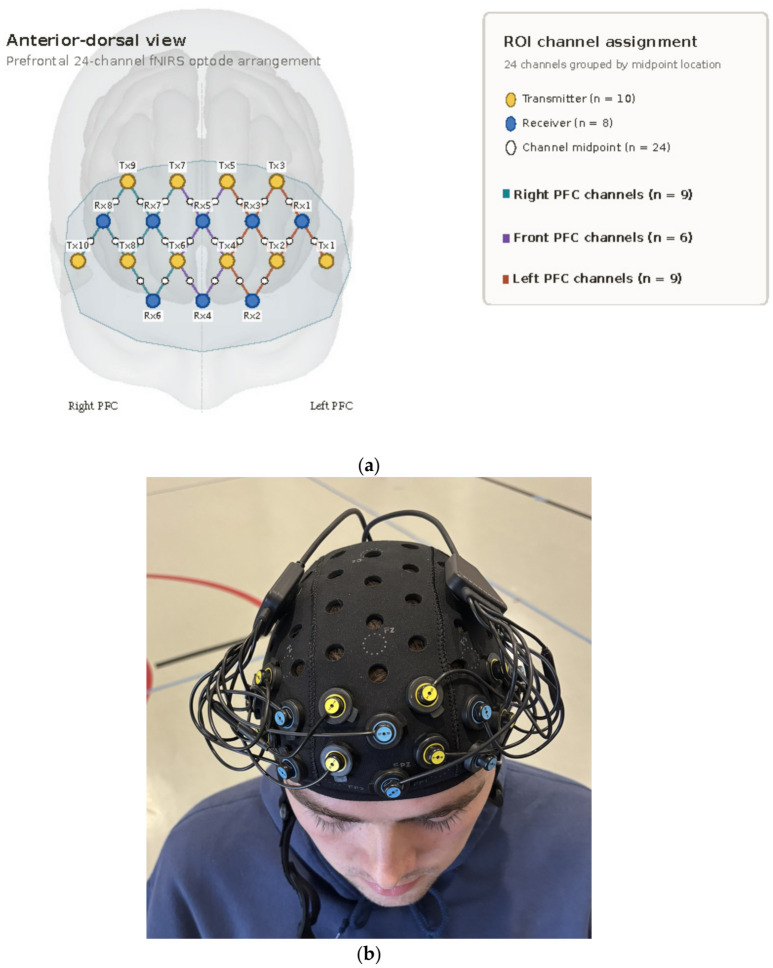
fNIRS system and prefrontal optode montage. (**a**) Anterior-dorsal view of the 24-channel prefrontal fNIRS montage. The montage included 10 transmitters and 8 receivers, yielding 24 source–detector channels positioned over the prefrontal cortex. Channels were grouped into predefined regions of interest based on channel midpoint location: left PFC, front PFC, and right PFC. (**b**) Example placement of the wearable fNIRS cap during data collection. fNIRS = functional near-infrared spectroscopy; PFC = prefrontal cortex; ROI = region of interest.

Concentration changes in oxygenated hemoglobin (oxy-Hb) in the prefrontal cortex (PFC) were measured using a portable, wearable fNIRS system, the Brite MKII (Artinis Medical Systems, Elst, The Netherlands), and are expressed in micromolar (μM) units. The system uses near-infrared light at two wavelengths to estimate changes in cortical hemoglobin concentration, and data were sampled at 50 Hz. Because the Brite MKII uses wireless Bluetooth technology, participants were able to complete the exercise protocols without restriction from wired connections. During data collection, participants wore the Brite MKII cap throughout the experimental visit. The optode montage consisted of 10 transmitters and 8 receivers, yielding 24 long-separation source–detector channels positioned over the PFC; short-separation channels were not used (Figure 2a,b).

Oxy-Hb was selected a priori as the primary hemodynamic outcome because it is commonly used as an index of exercise- and task-related cortical oxygenation and is often more sensitive to task-related changes than deoxy-Hb at the group level in fNIRS studies [8,19]. In the present study, deoxy-Hb and total-Hb were not analyzed as primary outcomes in order to limit the number of statistical comparisons and to focus on a single hemodynamic index aligned with our primary hypothesis regarding prefrontal oxygenation. The optode montage was kept consistent throughout the visit. Signal quality was visually inspected before data collection, and the optode montage was adjusted as needed to improve scalp contact and reduce poor signal detection. The 24 channels were grouped into three predefined PFC regions of interest (ROIs) based on channel midpoint location: left PFC, front PFC, and right PFC. The left and right PFC ROIs each included nine channels, whereas the front PFC ROI included six channels.

Only oxy-Hb signals were processed for the planned fNIRS analyses. Oxy-Hb values were baseline-corrected using the resting baseline recorded immediately before each condition. Raw signals were visually inspected for signal quality and motion artifacts, and channels with poor signal quality were excluded before ROI averaging. Because the present analysis used condition-level mean oxy-Hb values, preprocessing focused on obtaining stable baseline-corrected averages for each exercise bout. For analyses of oxy-Hb during exercise, the primary fNIRS outcome was mean baseline-corrected oxy-Hb averaged across the entire exercise bout. The exercise bout was not subdivided into temporal segments because the primary objective was to characterize overall prefrontal oxygenation during each exercise condition. For each ROI, oxy-Hb values were averaged across valid channels within that region. A global PFC oxy-Hb value was calculated as the average across all valid PFC channels and was specified as the primary fNIRS outcome. Regional ROI values were retained for secondary analyses to examine whether exercise-related oxy-Hb responses differed across left, front, and right PFC regions. For cognitive task analyses, oxy-Hb was averaged across the Flanker task for each condition and time point.

### 2.5. Flanker Task

Inhibitory control was assessed using a computerized arrow version of the Eriksen Flanker task implemented in the Psychology Experiment Building Language (PEBL) Test Battery [20,21]. The task required participants to identify the direction of a centrally presented target arrow while ignoring adjacent flanking arrows. Participants were instructed to respond as quickly and accurately as possible by pressing the left key when the central arrow pointed left and the right key when the central arrow pointed right.

**Figure 3:**
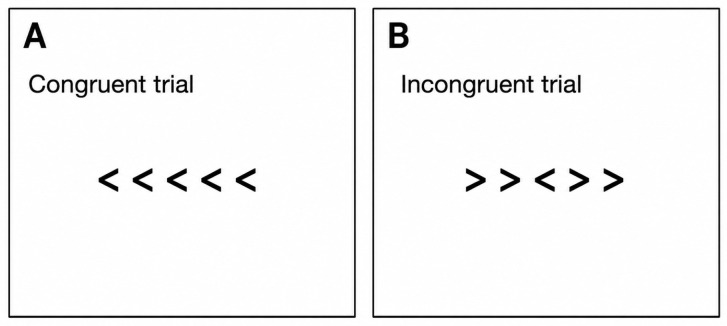
Example stimuli from the arrow version of the Eriksen Flanker task. Panel (**A**) shows a congruent trial, in which all arrows point in the same direction. Panel (**B**) shows an incongruent trial, in which the central target arrow points in the opposite direction to the flanking arrows. Participants responded based on the direction of the central arrow while ignoring the flanking arrows.

Each trial began with a white fixation cross presented for 500 ms, followed immediately by a horizontal array of five equally sized and equally spaced white arrows presented for 800 ms. The arrow array was 10.5 cm wide. In congruent trials, the four flanking arrows pointed in the same direction as the central target arrow, whereas in incongruent trials, the flanking arrows pointed in the opposite direction. For example, “< < < < <” and “> > > > >” represented congruent trials, whereas “< < > < <” and “> > < > >” represented incongruent trials, as illustrated in Figure 3. Participants completed 80 experimental trials at each assessment, consisting of 40 congruent and 40 incongruent trials presented in random order. The task included an equal number of left- and right-direction target responses. Before the experimental trials, participants completed 12 practice trials, which were not included in the analysis. The Flanker task lasted approximately 10 min.

Reaction time and response accuracy were recorded for each trial. Reaction time analyses were conducted using correct-response trials only. Trials with no response, anticipatory responses less than 200 ms, or slow responses greater than 1500 ms were excluded from all behavioral analyses. Reaction time outcomes were computed from the remaining valid-response trials, and accuracy and error outcomes were calculated based on the same set of valid trials.

The primary behavioral outcome was Flanker cost, calculated as mean reaction time on incongruent trials minus mean reaction time on congruent trials. Larger Flanker cost values indicate greater interference from task-irrelevant flankers, whereas smaller values indicate more efficient inhibitory control. Flanker cost improvement was calculated so that positive values reflected a reduction in interference from pre- to post-exercise. Mean reaction time, accuracy, total errors, congruent-trial performance, and incongruent-trial performance were analyzed as secondary behavioral outcomes. Reaction time outcomes were interpreted alongside accuracy and error measures to account for potential speed-accuracy trade-offs.

### 2.6. Statistical Analysis

Descriptive statistics were calculated for participant characteristics, exercise intensity measures, behavioral outcomes, and fNIRS outcomes. Continuous variables were summarized as mean ± standard deviation, and categorical variables were summarized as frequencies and percentages. Data distributions were inspected for outliers and normality before inferential analyses.

The primary behavioral outcome was Flanker cost improvement, calculated as pre-exercise Flanker cost minus post-exercise Flanker cost, such that positive values reflected a reduction in interference after exercise. The primary fNIRS outcome was mean baseline-corrected global PFC oxy-Hb during the exercise. Global PFC oxy-Hb was calculated as the average across all valid PFC channels.

The primary analysis examined whether global PFC oxy-Hb during exercise was associated with subsequent Flanker cost improvement. This association was tested using a linear mixed-effects model with Flanker cost improvement as the dependent variable, global PFC oxy-Hb during exercise as the primary predictor, exercise condition as a fixed effect, and participant included as a random intercept. A random slope was not included because each participant contributed only two condition-level observations. This model was selected to account for the within-subject design, in which each participant completed both aerobic and game-based exercise conditions.

Secondary analyses examined condition differences in exercise intensity, fNIRS outcomes, and behavioral outcomes. Because each participant completed both conditions, aerobic and game-based exercise were compared using paired-sample comparisons for mean heart rate, peak heart rate, accelerometer-derived activity intensity, RPE, global PFC oxy-Hb during exercise, and Flanker cost improvement. Secondary behavioral outcomes included total mean reaction time, accuracy, total errors, congruent-trial performance, and incongruent-trial performance.

Exploratory regional fNIRS analyses examined whether exercise-related oxy-Hb responses differed across predefined PFC ROIs. For these analyses, regional oxy-Hb values were analyzed using a 2 (condition: aerobic vs. game-based) × 3 (ROI: left, front, right PFC) repeated-measures model. These analyses were considered exploratory because the primary fNIRS hypothesis focused on global PFC oxy-Hb.

Order effects were evaluated in sensitivity analyses by adding randomized condition order to the primary mixed-effects model. If exercise intensity differed meaningfully between conditions, additional sensitivity analyses were conducted by including intensity measures as covariates. Statistical significance was set at *p* < 0.05. Effect sizes and 95% confidence intervals were reported where appropriate to support the interpretation of both statistically significant and non-significant findings.

## 3. Results

### 3.1. Participant Characteristics

Twenty-seven participants were enrolled in the study. Three participants were not included in the final analytic sample because of insufficient fNIRS signal quality, resulting in 24 participants included in the analysis. The analytic sample included 13 males and 11 females, with a mean age of 24.5 ± 3.7 years. Mean height was 67.4 ± 3.9 inches, mean body mass was 154.4 ± 27.7 lb, and mean body mass index was 23.7 ± 2.7 kg/m^2^. Participants reported an average of 187.6 ± 90.4 min/week of moderate-to-vigorous physical activity. Based on habitual activity classification, four participants were classified as low active, 15 as moderately active, and five as highly active. Condition order was fully counterbalanced, with 12 participants completing the aerobic exercise condition first and 12 completing the game-based exercise condition first (Table 1).

### 3.2. Exercise Intensity

Exercise intensity measures were examined to determine whether the aerobic and game-based exercise conditions were performed at comparable intensity. Mean heart rate did not differ significantly between the aerobic condition and the game-based condition, although values were descriptively slightly higher during game-based exercise: 124.1 ± 7.8 bpm versus 126.9 ± 6.7 bpm, respectively; MD = 2.8 bpm, 95% CI [−1.8, 7.4], *t*(23) = 1.24, *p* = 0.226, *d* = 0.25. Peak heart rate was nearly identical between conditions: 142.1 ± 8.2 bpm for aerobic exercise and 142.2 ± 8.9 bpm for game-based exercise; MD = 0.1 bpm, 95% CI [−5.3, 5.5], *t*(23) = 0.04, *p* = 0.971, *d* = 0.01.

Accelerometer-derived activity counts were also descriptively higher during game-based exercise but did not reach statistical significance. Mean ActiGraph counts were 2901.0 ± 472.9 counts/min during aerobic exercise and 3104.5 ± 503.6 counts/min during game-based exercise; MD = 203.5 counts/min, 95% CI [−54.6, 461.7], *t*(23) = 1.63, *p* = 0.116, *d* = 0.33. RPE ratings of perceived exertion were similar across conditions, with mean RPE values of 12.5 ± 1.2 for aerobic exercise and 12.9 ± 0.9 for game-based exercise; MD = 0.45, 95% CI [−0.29, 1.19], *t*(23) = 1.26, *p* = 0.219, *d* = 0.26 (Table 2). Overall, these measures indicated broadly comparable light-to-moderate exercise intensity across the two conditions; however, formal equivalence testing was not performed, and the conditions should be regarded as similar rather than strictly equivalent in intensity.

### 3.3. Flanker Task Performance

Flanker cost decreased from pre- to post-exercise in both conditions, indicating reduced interference after exercise. In the aerobic condition, Flanker cost decreased from 68.0 ± 19.5 ms before exercise to 60.7 ± 22.0 ms after exercise. This corresponded to a mean improvement of 7.2 ± 21.6 ms, 95% CI [−1.9, 16.3], *t*(23) = 1.64, *p* = 0.115, *d* = 0.33. In the game-based condition, Flanker cost decreased from 67.8 ± 19.8 ms before exercise to 47.6 ± 26.1 ms after exercise. This corresponded to a mean improvement of 20.2 ± 28.5 ms, 95% CI [8.2, 32.3], *t*(23) = 3.48, *p* = 0.002, *d* = 0.71. The direct paired comparison showed greater Flanker cost improvement after game-based exercise than after aerobic exercise; however, the between-condition difference did not reach statistical significance, MD = 13.0 ms, 95% CI [−0.6, 26.7], *t*(23) = 1.97, *p* = 0.061, *d* = 0.40.

Total mean reaction time decreased after both exercise conditions. In the aerobic condition, total mean reaction time decreased from 497.0 ± 46.0 ms to 483.1 ± 44.4 ms. In the game-based condition, total mean reaction time decreased from 492.9 ± 43.1 ms to 469.4 ± 43.0 ms. The reduction in total reaction time was significantly larger after game-based exercise than after aerobic exercise; MD = 9.6 ms, 95% CI [4.2, 15.1], *t*(23) = 3.68, *p* = 0.001, *d* = 0.75.

Accuracy remained high across assessments. In the aerobic condition, accuracy was 94.0 ± 3.6% before exercise and 95.8 ± 3.2% after exercise. In the game-based condition, accuracy was 94.1 ± 3.4% before exercise and 96.6 ± 2.7% after exercise. Error counts decreased from 4.8 ± 2.9 to 3.3 ± 2.6 errors in the aerobic condition and from 4.7 ± 2.7 to 2.8 ± 2.2 errors in the game-based condition. Accuracy did not decrease after either exercise condition.

Mean reaction time decreased more for incongruent trials than for congruent trials, particularly after game-based exercise. In the aerobic condition, congruent-trial reaction time decreased from 463.1 ± 40.9 ms to 452.7 ± 42.7 ms, whereas incongruent-trial reaction time decreased from 531.0 ± 52.5 ms to 513.5 ± 48.6 ms. In the game-based condition, congruent-trial reaction time decreased from 459.0 ± 39.6 ms to 445.6 ± 39.7 ms, whereas incongruent-trial reaction time decreased from 526.8 ± 48.5 ms to 493.1 ± 49.6 ms (Table 3).

### 3.4. Prefrontal Oxy-Hb During Exercise

Baseline-corrected global PFC oxy-Hb during exercise was significantly higher during game-based exercise than during aerobic exercise. Mean global PFC oxy-Hb was 0.122 ± 0.031 μM during aerobic exercise and 0.164 ± 0.043 μM during game-based exercise, where oxy-Hb values are expressed in micromolar (μM) units. The mean condition difference was 0.042 μM, 95% CI [0.019, 0.065], *t*(23) = 3.84, *p* < 0.001, *d* = 0.78.

**Figure 4:**
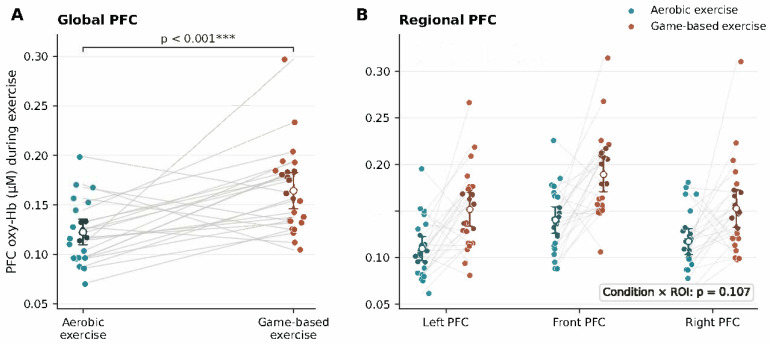
Global and regional prefrontal oxy-Hb during aerobic and game-based exercise. (**A**) Baseline-corrected global PFC oxy-Hb during aerobic and game-based exercise. Individual participant values are shown, with paired observations connected across conditions. (**B**) Regional PFC oxy-Hb during aerobic and game-based exercise across left, front, and right PFC regions of interest. Points represent individual participant values, and hollow circles indicate group means with error bars; asterisks indicate statistical significance (*** *p* < 0.001). PFC = prefrontal cortex; ROI = region of interest; oxy-Hb = oxygenated hemoglobin.

Exploratory ROI analyses showed that oxy-Hb differed across prefrontal regions and was higher during game-based exercise across ROIs. In the aerobic condition, baseline-corrected oxy-Hb values were 0.110 ± 0.031 μM in the left PFC, 0.141 ± 0.034 μM in the front PFC, and 0.117 ± 0.033 μM in the right PFC. In the game-based condition, values were 0.152 ± 0.043 μM in the left PFC, 0.189 ± 0.044 μM in the front PFC, and 0.153 ± 0.047 μM in the right PFC (Table 4). The repeated-measures model showed a significant main effect of condition, *F*(1,23) = 14.73, *p* < 0.001, and a significant main effect of ROI, *F*(2,46) = 85.73, *p* < 0.001. The condition × ROI interaction was not statistically significant, *F*(2,46) = 2.35, *p* = 0.107, suggesting that the game-based increase in oxy-Hb during exercise was not clearly localized to a single ROI (Figure 4).

### 3.5. Primary Association Between Oxy-Hb During Exercise and Flanker Cost Improvement

**Figure 5:**
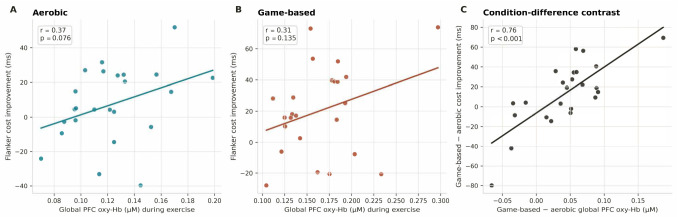
Association between global prefrontal oxygenation during exercise and Flanker cost improvement. Scatterplots show the association between global PFC oxy-Hb during exercise and Flanker cost improvement for the aerobic exercise condition, game-based exercise condition, and an exploratory condition-difference contrast. Flanker cost improvement was calculated as pre-exercise Flanker cost minus post-exercise Flanker cost, with positive values indicating reduced interference after exercise. The condition-difference contrast represents game-based minus aerobic values for both global PFC oxy-Hb during exercise and Flanker cost improvement. PFC = prefrontal cortex; oxy-Hb = oxygenated hemoglobin.

The primary analysis examined whether global PFC oxy-Hb during exercise was associated with subsequent improvement in inhibitory control. In the linear mixed-effects model, higher global PFC during exercise oxy-Hb was significantly associated with greater Flanker cost improvement. For interpretability, each 0.01 μM increase in PFC during exercise was associated with an estimated 3.71 ms greater improvement in Flanker cost, SE = 0.81, 95% CI [2.13, 5.30], *p* < 0.001. Expressed per 1.0 μM, the model coefficient was β = 371.49 ms, SE = 80.71, 95% CI [213.30, 529.69], *p* < 0.001 (Figure 5).

Exercise condition was not a significant independent predictor of Flanker cost improvement after accounting for global PFC oxy-Hb during exercise. The adjusted game-based versus aerobic condition effect was β = −2.56 ms, SE = 5.46, 95% CI [−13.26, 8.15], *p* = 0.640. This pattern suggests that the association between exercise and inhibitory control improvement was more closely related to individual differences in global PFC oxy-Hb during exercise than to exercise modality alone. As an exploratory analysis, we also examined a condition-difference contrast (game-based minus aerobic) to illustrate how between-condition differences in global PFC oxy-Hb during exercise corresponded to differences in Flanker cost improvement (Figure 5).

Sensitivity analyses produced similar results. The association between global PFC oxy-Hb during exercise and Flanker cost improvement remained significant after adding randomized condition order to the model, β = 3.68 ms per 0.01 μM, *p* < 0.001, and after separate adjustment for mean heart rate, accelerometer-derived activity counts, and RPE, all *p* < 0.001. Randomized condition order was not a significant predictor, *p* = 0.569.

## 4. Discussion

The present study examined whether global prefrontal oxy-Hb during exercise was associated with subsequent improvement in inhibitory control following intensity-monitored aerobic and game-based exercise in young adults. The primary finding was that greater global PFC oxy-Hb during exercise was associated with greater improvement in Flanker cost from pre- to post-exercise. Taken together, these findings indicate that a global prefrontal oxy-Hb response during exercise is related to subsequent changes in inhibitory control at the group level, but they do not specify the extent to which this response reflects cortical neural activity versus systemic cardiovascular or respiratory influences. This association remained evident after accounting for randomized condition order and after sensitivity analyses that considered exercise intensity indicators. In contrast, no significant condition difference was observed for the primary behavioral outcome. Thus, the main contribution of this study is not that game-based exercise produced clearly superior inhibitory control performance, but that prefrontal oxygenation during exercise was related to individual differences in subsequent inhibitory control improvement.

This finding extends current exercise–cognition research by suggesting that neurophysiological responses measured during exercise may provide information beyond traditional exercise intensity measures. Herold et al. proposed that fNIRS-derived brain parameters may be useful for understanding individual responses to exercise and may eventually contribute to more individualized exercise prescription approaches [22]. In the present study, global PFC oxy-Hb during exercise was not interpreted as a direct marker of superior neural activation, but as a physiological response that likely reflects a combination of cortical and systemic processes and that may be meaningfully associated with subsequent cognitive change. Thus, while higher global PFC oxy-Hb during exercise was associated with greater improvement in inhibitory control, this pattern should not be interpreted as evidence that increased oxy-Hb directly causes cognitive benefits. This distinction is important because acute exercise-related cognitive benefits are likely influenced by interacting neural, vascular, systemic, and task-related processes rather than by a single mechanism.

Game-based exercise elicited higher global PFC oxy-Hb during exercise than aerobic exercise. This finding is consistent with the view that open-skill or cognitively complex activities may impose greater attentional, visuomotor, response selection, and adaptive demands than more predictable closed-skill exercise [14,15,23]. In the present study, the pickleball-based activity required participants to monitor ball movement, coordinate body position, select responses, and adapt to a changing environment. These features may have contributed to the higher PFC oxy-Hb observed in the game-based condition. However, because mean heart rate and accelerometer counts were also higher, although not statistically different, in the game-based condition, the relative contributions of cognitive–motor engagement and systemic physiological responses to the observed oxy-Hb difference cannot be disentangled in this design.

However, the behavioral findings require a more cautious interpretation. Although Flanker cost decreased after both exercise conditions, the condition difference in Flanker cost improvement was not statistically significant. Therefore, the present results do not provide strong evidence that game-based exercise produced greater inhibitory control improvement than aerobic exercise. This interpretation is consistent with methodological discussions in acute exercise–cognition research emphasizing that cognitive effects are often modest [24] and can vary depending on exercise modality, timing of assessment, cognitive task characteristics, and participant-level factors [1,2,3]. Accordingly, the behavioral findings should be interpreted as evidence of post-exercise improvement across conditions, with insufficient evidence for a clear condition-specific advantage in the primary behavioral outcome. In particular, the significant within-condition improvement observed in the game-based condition, despite a non-significant between-condition comparison (*p* = 0.061), should be viewed as suggestive rather than definitive and should not be taken as proof that game-based exercise is superior to aerobic exercise.

Secondary behavioral outcomes provide additional context. Reaction time reductions were larger after game-based exercise, particularly for incongruent trials, whereas accuracy remained high across assessments. This pattern suggests that faster responses were not simply achieved at the expense of accuracy. Nevertheless, because Flanker cost improvement was specified as the primary behavioral outcome, these secondary findings should be interpreted as supportive rather than confirmatory. This distinction is important to avoid overstating the evidence for modality-specific cognitive benefits.

The fNIRS findings should also be interpreted carefully. Although oxy-Hb is commonly used as an index of cortical oxygenation, fNIRS signals recorded during movement and exercise may reflect both cortical hemodynamic responses and systemic physiological influences. Scholkmann et al. emphasized that fNIRS signals measured at the head include components related to neurovascular coupling as well as cardiorespiratory and autonomic systemic physiology [10]. Kirilina et al. further demonstrated that superficial physiological artifacts can contaminate forehead fNIRS signals and may contribute to false-positive interpretations if not adequately considered [11]. Therefore, higher PFC oxy-Hb during game-based exercise should not be interpreted automatically as greater or more beneficial neural activation.

The present study attempted to reduce this interpretive concern by monitoring exercise intensity using heart rate, accelerometry, and ratings of perceived exertion. The association between global PFC oxy-Hb during exercise and Flanker cost improvement remained evident after sensitivity analyses that considered intensity-related variables. This strengthens the interpretation that the primary association was not explained solely by gross differences in exercise intensity. However, these analyses cannot fully separate cortical oxygenation from extracerebral or systemic contributions. Future studies should incorporate more comprehensive physiological monitoring, including blood pressure, respiration, end-tidal carbon dioxide, and short-separation fNIRS channels when available, to better distinguish cortical hemodynamic responses from systemic artifacts [10,11,25].

The regional fNIRS analyses showed higher oxy-Hb during game-based exercise across left, front, and right PFC regions. Although the front PFC region showed the highest values overall, the condition-by-ROI interaction did not provide strong evidence that modality-related differences were localized to a specific PFC subregion. This supports the decision to treat global PFC oxy-Hb as the primary fNIRS outcome. Given the spatial resolution limits of fNIRS and the potential influence of systemic physiology during exercise, the non-significant condition × ROI interaction and the regional ROI findings should be regarded as exploratory and not as strong evidence for localization to a specific prefrontal subregion.

The present results also have implications for how game-based exercise is conceptualized in exercise–cognition research [26]. Game-based exercise should not be framed simply as a more enjoyable version of aerobic exercise, but as a movement context that combines physical exertion with cognitive–motor demands. Prior work suggests that game-based and open-skill activities may influence cognition differently from closed-skill exercise, although findings vary across populations, tasks, and intervention characteristics [12,14,15,16]. In the present study, game-based exercise produced higher PFC oxy-Hb during exercise, but this did not translate into a significant condition difference in the primary behavioral outcome. This pattern highlights the importance of distinguishing between neurophysiological responsiveness during exercise and measurable cognitive performance after exercise.

Several limitations should be acknowledged. First, the sample size was modest, and no formal a priori power analysis was conducted. Although the sample size is comparable to that of prior acute exercise–cognition fNIRS studies, it may have limited statistical power to detect small-to-moderate condition differences in Flanker cost improvement, and the present findings should be regarded as preliminary and partially exploratory. Second, both exercise conditions were completed during a single laboratory visit. Although the randomized, counterbalanced design and 10 min seated rest interval were used to reduce order and carryover effects, residual fatigue, learning, or carryover cannot be fully excluded, and the Flanker task was administered four times within one visit, introducing potential practice effects on reaction time. As a result, some portion of the observed post-exercise improvement in Flanker performance likely reflects task repetition and procedural learning rather than the acute effects of exercise per se. Consequently, the present design does not allow us to determine precisely how much of the improvement in inhibitory control can be causally attributed to the exercise bouts, and our inferences about exercise-specific effects should be interpreted with appropriate caution. Third, only oxy-Hb was analyzed. Although oxy-Hb is commonly used in fNIRS exercise–cognition research, future studies should consider deoxy-Hb, total hemoglobin, and systemic physiological measures to provide a more complete interpretation of hemodynamic responses. Fourth, oxy-Hb during exercise was summarized across the full exercise bout. This approach was consistent with the primary aim, but it may have obscured time-dependent changes in PFC oxygenation. Lastly, we did not formally assess participants’ prior pickleball experience, so unmeasured differences in familiarity or skill may have contributed to residual variability in gameplay during the pickleball-based condition.

Despite these limitations, the study has several strengths. The randomized, counterbalanced, within-subject design reduced between-person variability and allowed direct comparison of aerobic and game-based exercise under intensity-monitored conditions. The study also combined behavioral assessment with neurophysiological monitoring during exercise, addressing an important methodological gap in acute exercise–cognition research. Methodological guidance in this field emphasizes careful control of exercise dose, assessment timing, cognitive task selection, and transparent reporting of both significant and non-significant findings [1,2,19]. The present study contributes to this direction by focusing not only on whether acute exercise improves inhibitory control, but also on whether prefrontal oxygenation during exercise is associated with individual cognitive change.

## 5. Conclusions

In conclusion, greater global PFC oxy-Hb during exercise was associated with greater subsequent improvement in inhibitory control. Game-based exercise elicited higher PFC oxy-Hb during exercise than aerobic exercise, but no significant condition difference was observed for the primary behavioral outcome. These findings indicate that within-person variation in prefrontal oxygenation during exercise is related to acute exercise-related improvements in inhibitory control, but they do not establish a causal mechanism linking game-based exercise, oxy-Hb responses, and behavioral performance. Replication in larger samples and more comprehensive fNIRS and systemic physiological monitoring is needed before drawing mechanistic conclusions about whether oxy-Hb during exercise reflects beneficial cognitive engagement, systemic physiological load, neural efficiency, or a combination of these processes.

## Figures and Tables

**Table 1:** Participant characteristics and randomization.

Characteristic	Value
Sample size, n	24
Age, years	24.5 ± 3.7
Gender, female/male, n	11/13
Height, inches	67.4 ± 3.9
Body mass, lb	154.4 ± 27.7
BMI, kg/m^2^	23.7 ± 2.7
Weekly MVPA, min/week	187.6 ± 90.4
Activity level, n	High: 5; Low: 4; Moderate: 15
Dominant hand, right/left, n	23/1
PAR-Q screening	Cleared: 24
Randomized order (aerobic first/game-based first, n)	12/12

BMI = body mass index; MVPA = moderate vigorous physical activity; PAR-Q = physical activity readiness questionnaire. Data are presented as mean ± SD.

**Table 2:** Exercise intensity by condition.

Variable	Aerobic	Game-Based	Mean Difference [95% CI]	*p* Values
Mean heart rate (bpm)	124.13 ± 7.82	126.91 ± 6.71	2.78 [−1.84, 7.40]	0.226
Peak heart rate (bpm)	142.07 ± 8.15	142.17 ± 8.95	0.10 [−5.32, 5.51]	0.971
ActiGraph GT3X (counts/min)	2901.00 ± 472.92	3104.54 ± 503.61	203.54 [−54.60, 461.69]	0.116
RPE (6–20 scale)	12.47 ± 1.18	12.92 ± 0.92	0.45 [−0.29, 1.19]	0.219

Mean difference is game-based minus aerobic; RPE = rating of perceived exertion.

**Table 3:** Flanker behavioral outcomes.

Outcome	Aerobic Pre	Aerobic Post	Aerobic Change	Game-Based Pre	Game-Based Post	Game-Based Change	Between-Condition Difference [95% CI]	*p*	*d*
Flanker cost, ms	68.0 ± 19.5	60.7 ± 22.0	7.2 ± 21.6	67.8 ± 19.8	47.6 ± 26.1	20.2 ± 28.5	13.0 [−0.6, 26.7]	0.061	0.40
Total mean RT, ms	497.0 ± 46.0	483.1 ± 44.4	13.9 ± 10.3	492.9 ± 43.1	469.4 ± 43.0	23.6 ± 9.0	9.6 [4.2, 15.1]	0.001 *	0.75
Accuracy, %	94.0 ± 3.6	95.8 ± 3.2	1.8 ± 3.6	94.1 ± 3.4	96.6 ± 2.7	2.4 ± 2.6	0.6 [−1.2, 2.4]	0.485	0.14
Error count	4.8 ± 2.9	3.3 ± 2.6	1.5 ± 2.9	4.7 ± 2.7	2.8 ± 2.2	2.0 ± 2.1	0.5 [−1.0, 2.0]	0.485	0.14
Congruent RT, ms	463.1 ± 40.9	452.7 ± 42.7	10.3 ± 12.8	459.0 ± 39.6	445.6 ± 39.7	13.4 ± 14.8	3.1 [−5.6, 11.9]	0.465	0.15
Incongruent RT, ms	531.0 ± 52.5	513.5 ± 48.6	17.5 ± 16.7	526.8 ± 48.5	493.1 ± 49.6	33.7 ± 18.8	16.2 [7.5, 24.9]	<0.001 *	0.78

* Data are presented as mean ± SD; RT = reaction time. For reaction time, Flanker cost, and errors, change is calculated as pre minus post; positive values indicate a reduction after exercise. For accuracy, change is calculated as post minus pre and is expressed in percentage points.

**Table 4:** fNIRS Oxy-Hb outcomes during exercise.

Outcome	Aerobic	Game-Based	Mean Difference [95% CI]	*p*	*d*
Global PFC oxy-Hb, μM	0.122 ± 0.031	0.164 ± 0.043	0.042 [0.019, 0.065]	<0.001 *	0.78
Left PFC oxy-Hb, μM	0.110 ± 0.031	0.152 ± 0.043	0.042 [0.019, 0.065]	<0.001 *	0.79
Front PFC oxy-Hb, μM	0.141 ± 0.034	0.189 ± 0.044	0.048 [0.025, 0.072]	<0.001 *	0.86
Right PFC oxy-Hb, μM	0.117 ± 0.033	0.153 ± 0.047	0.035 [0.011, 0.060]	0.007 *	0.61

PFC = prefrontal cortex; oxy-Hb = oxygenated hemoglobin; μM = micromolar; * indicates *p* < 0.05.

## Data Availability

The data presented in this study are available on request from the corresponding author. The data are not publicly available due to ethical and privacy considerations associated with neural activity and physical assessment data.
